# Synthesis and characterization of monoacylglycerols through glycerolysis of ethyl esters derived from linseed oil by green processes

**DOI:** 10.1039/c9ra07834g

**Published:** 2020-01-13

**Authors:** Cristiane B. Hobuss, Felipe A. da Silva, Marco A. Z. dos Santos, Claudio M. P. de Pereira, Gracélie A. S. Schulz, Daniela Bianchini

**Affiliations:** Center of Science Chemistry, Pharmaceutical and Food, Federal University of Pelotas Pelotas RS Brazil gracelie.serpa@gmail.com +55 53 3275 7354 +55 53 3275 7356; Chemistry and Food School, Federal University of Rio Grande Rio Grande RS Brazil

## Abstract

The synthesis of monoacylglycerol (MAG) through the glycerolysis of ethyl ester mixture (biodiesel) was investigated in this study from linseed oil, low-cost alternative feedstock, using an alkaline catalyst with green reagent. The transesterification double step process (TDSP), reaction with ethanol to ethyl esters yielded 97%. In the glycerolysis reaction, the optimum operating condition was in a temperature of 130 °C with 5% sodium hydroxide (NaOH) in 1 : 5 biodiesel–glycerol and 12 h reaction time, in open reactor. The reaction conditions showed an interesting conversion and monoacylglycerol yield of 98% and 76%, respectively. The determination and characterization of reaction products was carried out by Gas Chromatography (GC) method, Infrared Spectroscopy (IR), Thermogravimetric Analysis (TGA) and Hydrogen Nuclear Magnetic Resonance Spectroscopy (^1^H NMR).

## Abstract 2

The synthesis of monoacylglycerol (MAG) through the glycerolysis of ethyl ester mixture (biodiesel) was investigated in this study from linseed oil, low-cost alternative feedstock, using an alkaline catalyst with green reagent.

## Graphical abstract

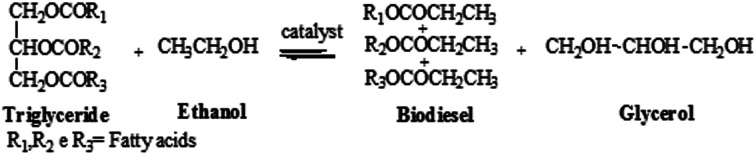

## 1. Introduction

Monoacylglycerols (MAGs) and their derivatives are widely used as emulsifiers in the food industry. They are considered safe and non-harmful agents at low concentration, giving no adverse reactions or toxicity to mucous membranes.^1^ In fact, MAGs can be found at low concentration in natural products as milk, for example.^2^ Besides the food industry, MAGs are applied in cosmetic products, such as ointments, lotions and deodorants.^1^ In the plastic industry, MAGs work as antistatic, lubricant and plastifying agents.^3^ In the textile industry, MAGs can improve color fastness and contrast in fabrics.^1^

MAGs are esters of the glycerol, a trihydric alcohol in which only one of the hydroxyl groups (OH groups) is esterified with a long-chain fatty acid.^4^ MAGs can be prepared by glycerolysis of triglycerides from fats or vegetable oils,^5,6^ by the glycerolysis of alkyl esters,^5,7,8^ by the esterification of fatty acids with glycerol and by transesterification of oils with short chain alcohols.^9^ Industrial glycerolysis process are usually promoted with fats or oils using homogeneous alkaline catalysts, such as NaOH, KOH and Ca(OH)_2_, at elevated temperatures (*e.g.*, 255 °C) giving rise 40–60% of MAGs. However, high temperatures can affect the physicochemical properties of final product and its application.^5–7^

He *et al.*,^9^ reported the synthesis of MAGs with high yield *via* one-step enzymatic ethanolysis of commercial edible anchovy oil. The authors reported that MAGs with high content of *n* − 3 polyunsaturated fatty acids (*n* − 3PUFAs) can be obtained by using *Candida antarctica* lipase A (CAL-A) enzyme.^9^ Although MAGs can be obtained at lower temperatures with enzymatic catalysts, long reaction times are required to obtain MAGs with high yield. Besides, enzymes are more expensive than usual chemical catalysts. Thus, from the industrial point of view the enzymatic route is not economically viable. Lozano *et al.*,^10^ suggested the biocatalytic synthesis of MAGs, out by the direct esterification of fatty acids (*i.e.* capric, lauric, myristic, palmitic and oleic acids, respectively) with glycerol, catalyzed by lipase Novozym 435 in different ionic liquids (ILs), as an alternative method to industrial glycerolysis process, a high selectivity of MAGs was observed with the 1-dodecyl-3-methylimidazolium tetrafluoroborate.^10^ IL is considered a green solvent and promising catalyst, presenting high thermal stability with the possibility of recovery and reuse. However, IL presents high cost, complex preparation and some toxicity. Schulz *et al.*,^8^ obtained 72% of MAGs performing a simple and direct glycerolysis from methyl esters and glycerol. The authors performed the glycerolysis with alkaline catalysts, lower reaction times and mild conditions of temperature (<150 °C). Glycerolysis reactions performed with alkyl esters occur at temperatures lower than that performed with triacylglycerols (TAG). Besides, alkyl ester glycerolysis is usually faster than that performed with TAGs.^8^

Biodiesel which is recognized as “green fuel”, is alternative fuel,^11^ consists of fatty acid methyl esters (FAMEs) or fatty acid ethyl esters (FAEEs). It can be obtained from animal fat or vegetable oils through transesterification reactions of large TAGs,^12,13^ or through esterification reactions of free fatty acids (FFAs).^14^ The main source of TAGs for biodiesel production are vegetable oils from sunflower, tobacco seed, rapeseed, corn, palm, linseed, castor, babaçu, soybean, peanut and jatropha, among others.^15,16^ The oil content in oilseeds strongly depends on the characteristic of each crop and the environmental conditions.^17^ In Brazil, for instance, the climatic and soil conditions are convenient for the production of linseed (*Linum usitatissimum*) crop.^18,19^

According to the Table 1, the linseed oil is a bioactive ingredient with high α-linolenic acid content.^21,22^ The long-chain polyunsaturated fatty acids (LC-PUFAs), especially linoleic acid (C18:2*n* − 6) and linolenic acid (C18:3*n* − 3) have anticarcinogenic and cardioprotective roles in humans. The reduction of the consumption of saturated fatty acids (SFAs) and the increase of the consumption of polyunsaturated fatty acids (PUFAs), fatty acids with more than one double bond are encouraged. Monounsaturated fatty acids (MUFAs, fatty acids with one double bond) are also usually regarded beneficial for human health.^23^ Linseed seeds contain high amount of oil, around 40%, which can be converted into biodiesel.^18,19^

**Table 1:** Fatty acid composition of linseed oil^20^

Name of fatty acid^a^	Structure	Formula	Weight (%)
Palmitic	C16:0	C_16_H_32_O_2_	5
Stearic	C18:0	C_18_H_36_0_2_	3
Oleic	C18:1	C_18_H_34_O_2_	21
Linoleic	C18:2	C_18_H_32_O_2_	15
Linolenic	C18:3	C_18_H_30_O_2_	54

a Other fatty acids add up to approximately 2%.

Transesterification reactions of TAGs can be performed with homogeneous or heterogeneous catalysts, in acidic or basic medium.^24,25^ Lipases and ion exchange resins can also catalyze these reactions.^26,27^ Reactions catalyzed by bases as NaOH, KOH or alkoxides can be performed faster than that catalyzed by acids. Another advantage of basic catalysis is the possibility to carry out the transesterification reactions at low temperatures, near alcohol boiling point.^14,28^ On the other hand, the transesterification reactions catalyzed with basis is sensitive to water content in the raw matter. Even low content of water in TAGs results in the soap formation, which decreases the efficiency of the biodiesel synthesis. Samios *et al.*,^29^ proposed a two-step synthesis with KOH as basic catalyst in the first step, followed by addition of H_2_SO_4_ as acidic catalyst in the second step. This methodology was denominated Transesterification Double Step Process (TDSP). The combination of catalysts results in high conversion efficiency, excellent biodiesel quality and easy phase separation procedure between biodiesel and glycerol. Besides, the synthesis of biodiesel by this method is faster than that performed with conventional methods.^29^

The nature of primary alcohol is also an important parameter in the biodiesel synthesis. Acid-catalyzed transesterification reactions have included methanol, ethanol, propanol, butanol, and amyl alcohol. Methanol and ethanol are used most frequently in both laboratory research and the biodiesel industries.^30,31^ Methanol presents the best performance in the biodiesel synthesis and can be obtained with high purity, which makes it the first choice for the transesterification reactions. However, methanol is a toxic compound to human health and its employment in the synthesis of biodiesel to produce MAGs is not acceptable for food industry.^14^ The employment of ethanol instead of methanol offers a number of benefits, include higher miscibility with vegetable oils that allows better contact in the reaction step.^32^ Besides, as reduce environmental impacts avoiding the emission of greenhouse gases and using a renewable product obtained by biotechnological processes, the ethanol still is a non-toxic compound and safe to human health,^33^ and it is produced in large quantities from sugar cane in Brazil.^36^

Schulz *et al.*,^8^ reported the synthesis of MAGs from glycerolysis of the fatty acid methyl esters with glycerol. These FAMEs were obtained from transesterification reactions of the linseed oil and methanol.^8^ Despite the advantages of MAGs obtained from FAMEs and glycerol reported by Schulz *et al.*^8^ Methanol is highly toxic and non renewable as it is mainly derived from non renewable sources such as petroleum refining products.^34^ The use of methanol in the synthesis restricts the use of MAGs for food industries. Methanol traces are not desired in food and other products for human consumption.^35^ Benefits of MAGS for human health would be best exploited if the MAGs would be synthesized from FAEE and glycerol. Guzatto *et al.*,^35^ performed transesterification reactions of several vegetable oils with ethanol. FAEEs were successfully synthesized with high yield *via* TDSP process.^35^

In this context, this work aims to optimize the synthesis of MAGs from fatty acid ethyl esters (FAEE) obtained by the transesterification reactions of linseed oil and ethanol. The glycerolysis process proposed in this work rises as an alternative to reduce the environmental impact of the large amount of glycerol produced in the biodiesel synthesis by fuel industries,^37^ it is expected that this quantity will increase in the future due to the growing demand for biodiesel.^38^ This methodology uses mild reaction conditions and non-toxic solvent for MAGs synthesis. In addition, the starting material of glycerolysis (biodiesel) comes from transesterification reaction performed with green reagent (ethanol). Therefore the process is sustainable because all products generated and surplus components can be recovered and reused in the process. The final product is safe to be applied in food, beverage and pharmaceutical industries.

## 2. Materials and methods

### 2.1. Materials

Chemicals were purchased as follows: purified linseed oil (Mundo dos Óleos, Brasília, Brazil); glycerin, KOH, NaCl and anhydrous Na_2_SO_4_ (Synth, Diadema, Brazil); NaOH and *n*-heptane (Vetec, Duque de Caxias, Brazil); anhydrous ethyl alcohol and H_2_SO_4_ (Dinâmica, Indaiatuba, Brazil); deuterated acetone from Scielab. The internal standard for CG measurements tricaprin (1,2,3-tricapropylglycerol), the external standards (monolein, diolein and triolein), and the derivates *N*-methyl-*N*-(trimethylsilyl)trifluoroacetamide (MSTFA) were purchased from Sigma-Aldrich, (USA) with 99.9% purity.

### 2.2. Experimental setup

#### 2.2.1. Transesterification reaction: production of biodiesel from linseed oil

The obtaining of ethyl esters from linseed oil was carried out in two steps, produced according to the TDSP method, methodology was adapted from Guzatto *et al.*^35^ The first stage consisted of basic catalysis using KOH, followed by acid catalysis with H_2_SO_4_.

Initially, 120 mL of ethanol was introduced in a simple reactor equipped with a reflux device and stabilized at 65 °C. Potassium hydroxide (2.0 g) was added to the ethanol, and the mixture was vigorously stirred until the potassium hydroxide was completely dissolved, allowing the formation of the active species of basic catalyst. Under constant stirring, 100 mL of linseed oil were then added to the reaction vessel. The alcohol/oil/catalyst molar ratio was 20 : 1 : 0.35. The system was remained under these conditions for 30 min. In the second step, 4 mL of sulfuric acid (P.A., 18.77 mol L^−1^) was added dropwise to the reaction mixture, followed by soft heating until 80 °C and the addition of 60 mL of ethanol. After stabilizing temperature the system was remained under these conditions for 2 h 30 min. Reaction mixture was filtered to remove the solid residue (K_2_SO_4_). Liquid phase was concentrated using a rotary evaporator to remove the alcohol excess and this specific procedure promoted a fast and clear separation in two liquid phases. The ethyl esters (biodiesel) were in the upper phase, with some traces of non-reacted oil, monoacylglycerols, diacylglycerols and a small amount of ethanol. The glycerol formed was in the lower phase. After phase separation, the biodiesel was washed with NaCl solution (5%) and dried under anhydrous Na_2_SO_4_ followed by filtration procedure. Obtained ∼87 mL from the product.

#### 2.2.2. Glycerolysis reaction: synthesis of acylglycerols

The glycerolysis reactions were performed by varying the reaction time (6–12 h) and temperature (100–150 °C). The biodiesel/glycerol/catalyst molar ratio was 1 : 5 : 0.38. The excess of glycerol was added in order to shift the equilibrium to a greater production of MAGs.

Initially, 30 g of biodiesel, 46 g of glycerol and 1.5 g of NaOH were introduced in a simple reactor and the mixture was constantly stirred. The glycerolysis was performed under reflux or in open system with variable time and temperature. The product obtained is a viscous liquid, this was washed with NaCl solution (5%) and heated at 70 °C. The heating favors the solubilization of the glycerol in the aqueous solution. The product was subsequently cooled in an ice bath, to facilitate removal of the aqueous phase by loading glycerol. This washing process with heating and cooling was performed several times, after the product was dried by heating at 100 °C. Obtained ∼20 g from the product.

### 2.3. Analytical procedures

The ^1^H NMR spectra were obtained by spectrometer Bruker instrument operating at 400 MHz. The linseed oil and biodiesel samples were dissolved in deuterated chloroform and the glycerolysis product in deuterated acetone. Chemical shifts are related in parts per million (ppm) relative to the internal TMS standard.

The nature of the samples were analyzed by using a Shimadzu spectrometer (IRAffnity-1, Kyoto, Japan), coupled to an Attenuated Total Reflectance accessory (ATR) (Pike Tech, Madison, WI.). The samples were placed on the zinc selenide (ZnSe) crystal and the analyses were performed in the range of 4000–700 cm^−1^, co-adding 32 scans and spectral resolution of 4 cm^−1^.

Thermal stability of samples was obtained using a Shimadzu thermogravimetric analyzer (DTG-60, Kyoto, Japan). Samples (4–6 mg) were heated between 25 and 650 °C at a heating rate of 10 °C min^−1^ and a nitrogen gas flow of 50 mL min^−1^.

A Shimadzu GC-2010 chromatograph equipped with a flame ionization detector (FID) was used for individual separation and determination of FAEE. The analysis of the samples was carried using the standard technique ASTM 6584, is used to determine the content of free glycerin, monoacylglycerol, diacylglycerol and triacylglycerol in fatty acid methyl esters, which was adapted to quantify the conversion of ethyl esters into reaction products, yield and selectivity. A 15 m × 0.32 mm × 0.1 μm SGE-HT5 column was used for the determination of FAEE. Chromatographic conditions were: detector temperature: 380 °C; injector temperature: 50 °C; gas carrier: nitrogen; linear gas velocity: 12 cm s^−1^; total running time: 40 min; oven temperature program: 50 °C during 1 min, 15 °C min^−1^ until 180 °C, 7°C min^−1^ until 230 °C, and 10 °C min^−1^ until 370 °C, remaining isothermally for 10 min. Three solutions were used for quantitative analysis of glycerolysis products: a standard solution (solution 3) used to determine retention time and calculate the response factor in relation to the internal standard, a solution containing the biodiesel (with the biodiesel used in the reaction) and a solution containing the products (solution with glycerolysis products), according to technique, dissolved in heptane. Aliquots of 1 μL of these solutions were injected into the equipment. This methodology was used by Schulz *et al.*^8^

## 3. Results and discussion

Different analyses were carried out to characterize biodiesel and monoacylglycerol synthesized from linseed oil. The oil conversion to biodiesel was evaluated using ^1^H NMR and the biodiesel conversion to MAG was evaluated using ^1^H NMR and CGFID, following the methodologies described in the Section 2.2. The MAG chemical structure was identified by ^1^H NMR and FTIR. Thermal stability of the biodiesel and MAG was evaluated by TGA.

### 3.1. The transesterification process

The transesterification reactions of linseed oil to FAEE (biodiesel) were performed by TDSP methodology and in the presence of the green reagent ethanol.

**Fig. 1:**
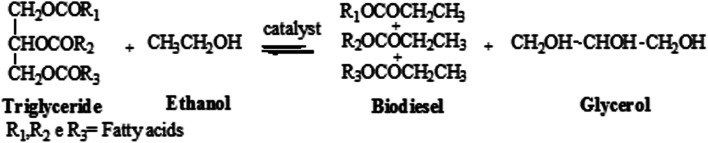
Scheme of the transesterification reaction.

The scheme of the transesterification reaction is shown in the Fig. 1.

Molar ratio of alcohol to oil is one of the most significant factors affecting the conversion efficiency and yield of biodiesel, the molar ratio of alcohol to oil is 3 : 1 and the reaction is reversible, higher molar ratios are required to increase the miscibility and to enhance the contact between the alcohol molecule and the triglyceride. Therefore, and an excess of ethanol should be added in order to shift thermodynamic equilibrium towards the formation of biodiesel.

**Fig. 2:**
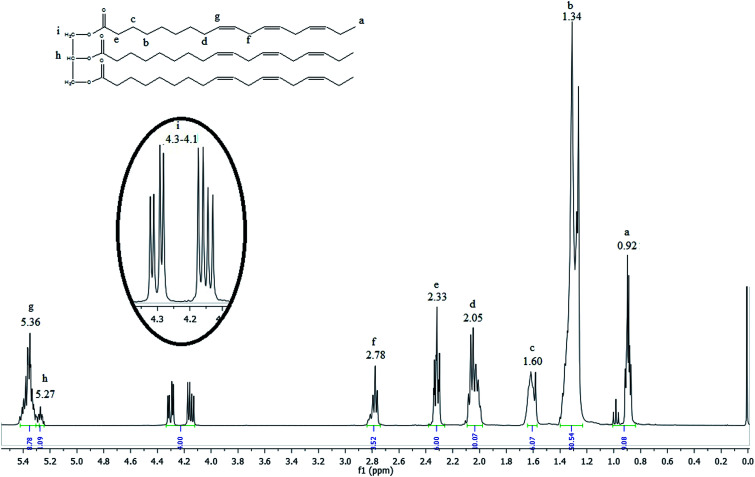
^1^H NMR spectrum of the linseed oil.

The ^1^H NMR spectrum of the linseed oil (Fig. 2) emphasizes the chemical shift regions of interest.

The most shielded peak observed at 0.92 ppm (peak a) is characteristic of the hydrogen atoms of terminal methyl groups in fatty acid chains, as indicated in the linseed oil chemical structure inserted in the Fig. 2. The signals observed between 2.78 and 1.34 ppm (peak f to b) are attributed to hydrogen atoms of internal methylene groups in fatty acid chains. The olefinic hydrogen atoms of carbon–carbon double bonds of fatty acids are placed in a downfield region at 5.36 ppm (peak g). The highlighted signals located between 4.1 and 4.3 ppm (peak i) are characteristic of external hydrogen atoms of glycerol fragment. The internal hydrogen atom of glycerol fragment is observed at 5.27 ppm (peak h).^35^

**Fig. 3:**
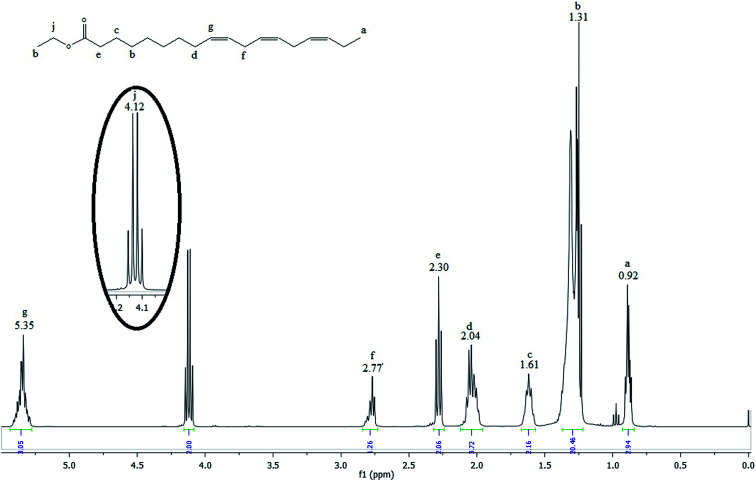
^1^H NMR spectrum of the linseed oil biodiesel.

Fig. 3 shows the ^1^H NMR spectrum of the linseed oil biodiesel.

Many signals in the biodiesel spectrum are similar to those observed in the linseed oil spectrum. However, glyceridic fragment was removed during transesterification reactions and replaced by an ethyl ester fragment. Then, the absence of the peaks h and i in the spectrum of the Fig. 3 indicates that linseed oil was completely converted to biodiesel. The hydrogen atoms of methylene group of ethyl ester, highlighted in the Fig. 3, can be observed as a quartet at 4.12 ppm (peak j). According to Guzatto *et al.*,^35^ the hydrogen atoms of methyl group of ethyl ester are overlapped by other signals at 1.2 ppm. These signals appear around 1.3 ppm (peak h) in the spectrum of the Fig. 2.

Gelbard *et al.*,^39^ Knothe *et al.*, Kenar *et al.*,^40^ Morgenstern *et al.*,^41^ and Tariq *et al.*,^42^ have used a reliable methodology to obtain ethyl ester conversion (*C*_EE_) from mono-, di- and triacylglycerols, as well as fatty acids methyl esters. This methodology is based on the integration of some peaks of ^1^H NMR spectrum of biodiesel. In the Fig. 3, the peak at 4.12 ppm was used to calculate the ethyl ester content. The peak at 2.30 ppm was chosen to represent all possible linseed oil derivatives and all other species that were formed during transesterification reactions. According to eqn (1):1
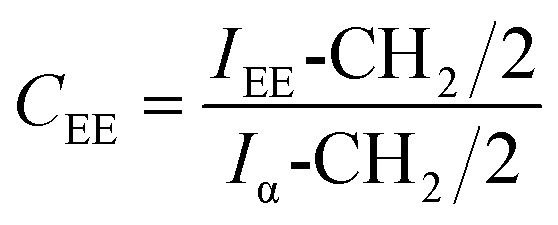
*I*_EE_-CH_2_ is the integration value of the peak j, which corresponds to the hydrogen atoms of methylene of ethyl ester. *I*_α_-CH_2_ is the integration value of the peak e, which is attributed to the hydrogen atoms of methylene adjacent to carbonyl group. The factor 2, which divides both integration values, is the normalization factor, because each integral corresponds to two hydrogenes.^35^

Thus, the transesterification reaction performed in this work produced 97% of biodiesel from the linseed oil. The present yield was similar to the 98% obtained by Guzatto *et al.*,^35^ and higher than the 85% obtained by Schulz *et al.*,^8^ considering a high conversion efficiency using a non-toxic reagent.

### 3.2. The glycerolysis process

In the present work, the glycerolysis reactions of ethyl esters were performed in basic medium at different temperatures and reaction times.

**Fig. 4:**
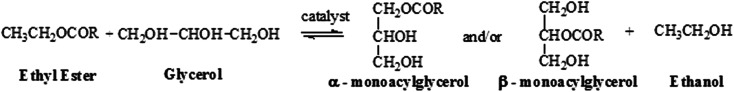
Scheme of the ethyl ester glycerolysis reaction.

The scheme of the glycerolysis reaction is shown in the Fig. 4.

Glycerolysis is a reversible reaction and an excess of glycerol should be added in order to shift thermodynamic equilibrium towards the formation of MAGs. In this work, reactions were performed with molar ratio biodiesel/glycerol of 1/5, since Schulz *et al.*,^8^ have reported that it is the most efficient molar ratio.

The Fig. 4 shows that the byproduct of the glycerolysis reaction is ethanol, another advantage over the work done by Schulz *et al.*, in which the reaction byproduct is methanol, thus reducing the environmental impact with a less toxic residue generated.

GC analyses were used to quantify the glycerolysis products, according to ASTM method D6584. Chromatograms of the biodiesel before conversion, the glycerolysis product and the mixture of internal and external standards were used to evaluate the biodiesel conversion and the respective acylglycerols yield.

In order to quantify glycerolysis products, the response factors (rF) of each involved specie, should be considered. Biodiesel weight conversion (*C*_BD_) can be obtained from areas of chromatogram peaks of the biodiesel before conversion and the glycerolysis products, according to eqn (2):2
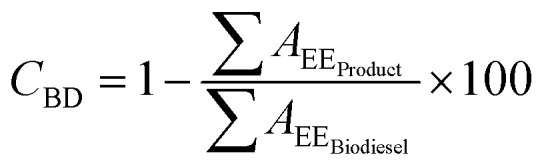
∑*A*_EE_Biodiesel__ is the summation of the peak areas corresponding to ethyl esters of the biodiesel before the reaction. ∑*A*_EE_Product__ is the summation of the peak areas corresponding to ethyl esters in the product after the reaction.

The acylglycerols yield (%) must be expressed in relation to the *C*_BD_. The mono-, di-, and triacylglycerol yield (%) (*Y*_MG_, *Y*_DG_, and *Y*_TG_, respectively) were calculated according to eqn (3)–(5):3
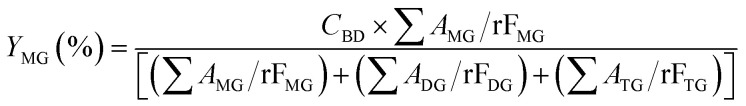
4
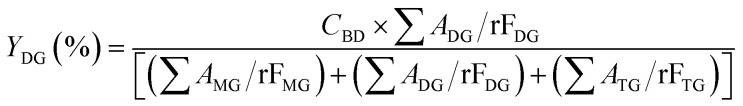
5
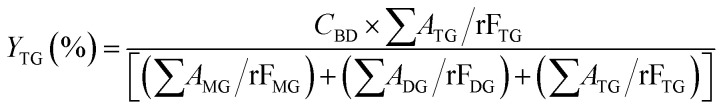
*A*_MG_, *A*_DG_, and *A*_TG_ are the respective areas. Term rF gives the corresponding response factors.

**Fig. 5:**
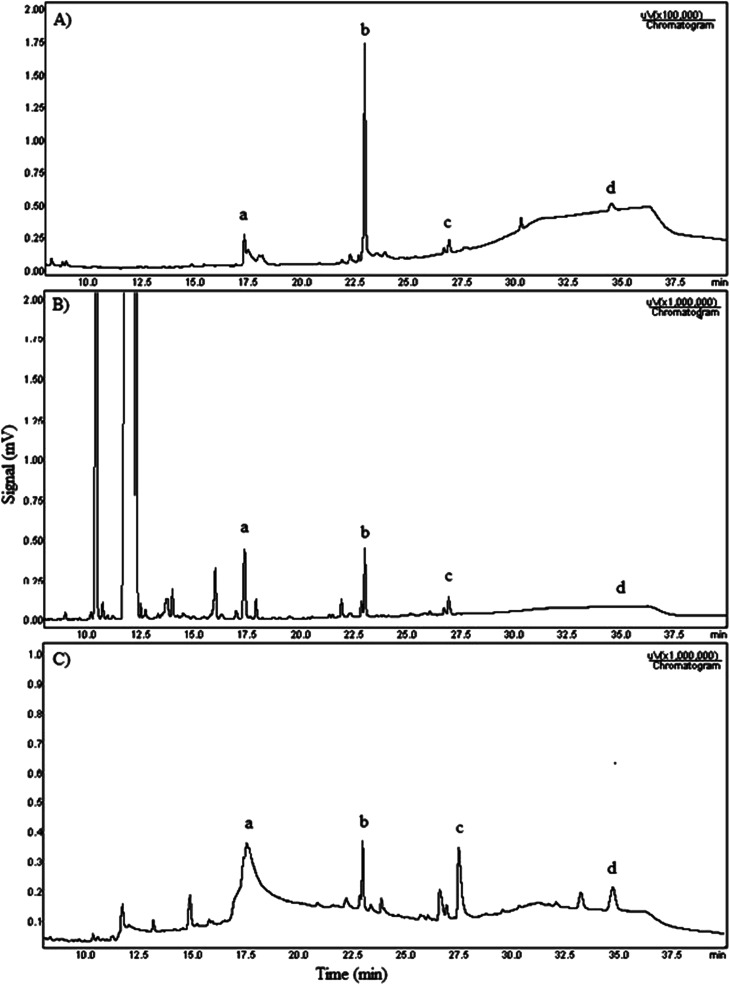
Chromatograms of the: (A) external and internal standards mixture: nonolein (a), tricaprin (b), diolein (c) and triolein (d); (B) pure biodiesel before the glycerolysis reaction; (C) glycerolysis product obtained with 76% yield of MAG.

The Fig. 5 shows the three chromatograms used to quantify the biodiesel conversion and glycerolysis products yield.

According to chromatogram obtained, that ethyl esters eluted between 10 and 16 min retention time. Monoacylglycerols (a) appear in the range 17 min, diolein (c) appears after 27 min and triolein (d) is detected in chromatogram after 35 min. The chromatogram A is the one composed with the external standards monoolein, diolein and triolein. The chromatogram B is the biodiesel before reaction. The chromatogram C is the sample prepared with the product obtained in the test using 5% NaOH, biodiesel : glycerol 1 : 5 ratio and 12 h time. All three chromatograms include internal standard tricaprin (b) at the same concentration.

In order to improve the performance on the biodiesel conversion to MAG the effects of temperature and reaction time have been studied to the glycerolysis reactions.

According to the Table 2 a simple comparison of these data indicates that the highest value of the biodiesel conversion, 98%, was obtained for the experiment performed with reaction time of 12 h at 130 °C, in open reactor (test M5). For open reactor testing, the best *C*_BD_ results are obtained when temperature and reaction time are increased simultaneously. At the lowest temperature (test M7, which has a long time) and at the shortest time (test M4, at a high temperature) the results were worse. Simultaneously increasing both factors seems to improve conversion. However, a temperature increase above 130 °C (see test M6) does not appear to improve biodiesel conversion and the energy cost of the reaction is higher, which is not interesting from an industrial point of view. All tests performed with open system were more effective than the reflux system due to the evaporation of ethanol, a volatile by-product of the glycerolysis reaction. The ethanol removal shifts thermodynamic equilibrium towards the MAG formation. In a glycerolysis industrial plant the ethanol could be recovered at the end of the process, which would avoid waste generation, one of the basic principles of green chemistry.^43^

**Table 2:** Biodiesel conversion (*C*_BD_) values obtained according to the parameters used in the glycerolysis reaction^a^

Reaction	Time (h)	Temp. (°C)	*C*_BD_ c (%)	MAG (%)	DAG (%)	TAG (%)
M1^a^	10	130	50	44	1	5
M2^b^	10	130	88	52	7	28
M3^b^	8	130	89	39	11	39
M4^b^	6	130	76	49	5	23
M5^b^	12	130	98	76	7	15
M6^b^	12	150	98	22	27	48
M7^b^	12	100	84	26	8	49

a Reflux.

b Open.

c *C*_BD_: converted biodiesel.

It should be considered that the aim of this study is optimize conditions for MAGs synthesis. In other words, it means obtain the maximum yield in monoacylglycerol, and a process with as mild temperatures as possible is sought. The Table 2 shows that higher content of MAG can be obtained with reaction time of 12 h at 130 °C (test M5). Glycerolysis reaction temperature seems to be an important parameter to reach higher MAG content. Experiments performed at 100 and 150 °C presented a lower contend of MAG and an increasing content of DAG and TAG in the glycerolysis products.

It is noteworthy, the great difficulty of mass transfer, due to the high viscosity of the product, therefore, to remove excess glycerol from the formed product, a slight heating to 70 °C is required, as explained in Section 2.2.2, and subsequent cooling with ice bath to facilitate phase separation.

Thus, these results demonstrate the feasibility of producing MAGs at relatively low temperature in biodiesel glycerolysis reactions. These good results can be explained by the ethyl esters that are more miscible in glycerol when compared to the vegetable oil, used in the traditional method of glycerolysis by industry, with temperatures above 200 °C. These results are similar to those observed by Schulz *et al.*,^8^ which also observed good results with 130 °C, in the present work, the results were as good as those of the article, but with green reagent.

**Fig. 6:**
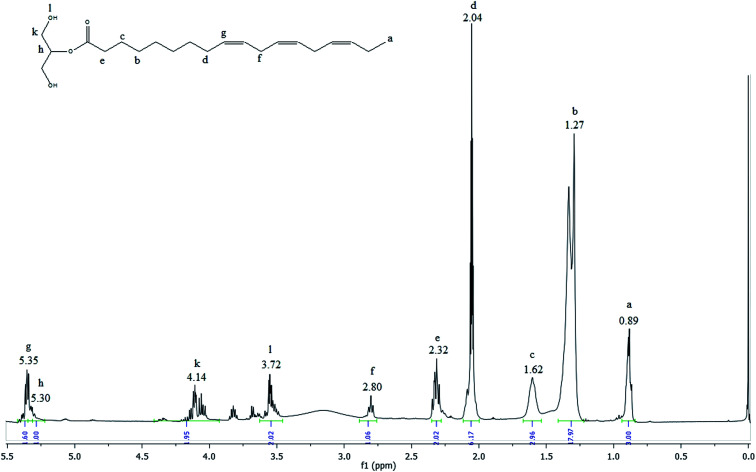
^1^H NMR spectrum of the glycerolysis product obtained with 76% yield of monoacylglycerol in green solvent.

The ^1^H NMR spectrum of MAG is shown in the Fig. 6.

The most shielded peak observed at 0.89 ppm (peak a) is characteristic of the hydrogen atoms of terminal methyl groups in fatty acid chains. The signals observed between 2.80 and 1.27 ppm (peak f to b) are attributed to hydrogen atoms of internal methylene groups in fatty acid chains. The olefinic hydrogen atoms of carbon–carbon double bonds of fatty acids are placed in a downfield region at 5.35 ppm (peak g). The small signal observed at 5.30 (peak h) included in this area is originated from the central glycerol hydrogen. The acylglycerol protons signal at 4.14 ppm (peak k) are characteristic of external hydrogen atoms of glycerol fragment and the signal at 3.72 (peak l) is attributed to hydrogen of the hydroxyl group. The peaks k and l are characteristic of the monoacylglycerols.^44^ Comparing the spectra presented in Fig. 2, 3 and 6, it is observed that the signal referring to the double bonds were not changed, which means that after the reaction process the MAG maintains the double bonds, which characterize important and beneficial for the human health and can be used in the food and pharmaceutical industry.

**Fig. 7:**
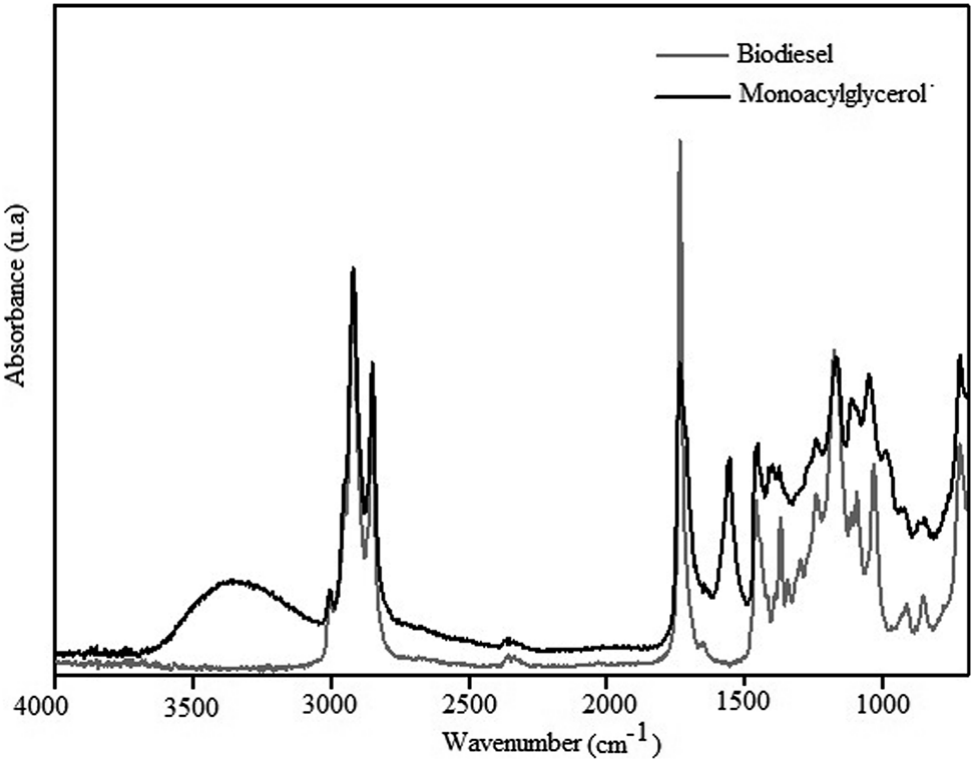
IR spectrum of the monoacylglycerol synthesized from linseed oil biodiesel.

The IR spectrum of MAG obtained from linseed oil biodiesel, with 76% yield, is shown in Fig. 7.

**Fig. 8:**
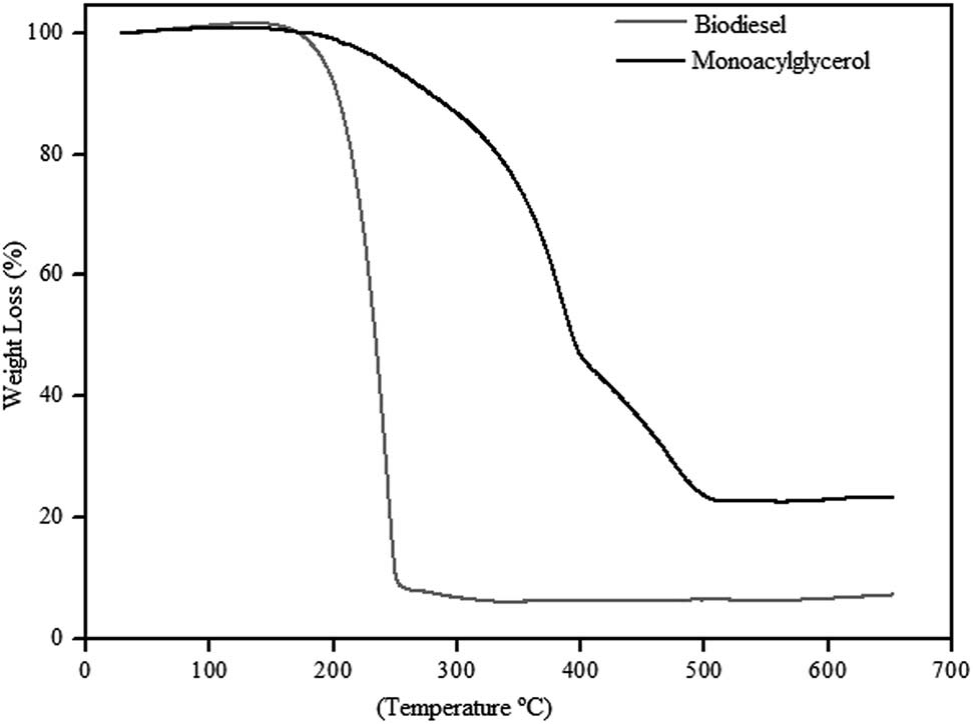
TGA curves of linseed oil biodiesel and monoacylglycerol.

The bands shown in the spectrum of the Fig. 7, assigned according to Schulz *et al.*,^8^ confirm the formation of MAG. The broad and strong band centered at 3360 cm^−1^ was assigned to O–H stretching modes, *ν*(O–H), typical of hydroxyl groups remaining from glycerolysis reaction of biodiesel. This enlargement suggests a large extension of OH groups associated by H-bonds. The weak band at 3008 cm^−1^ can be assigned to C–H stretching modes, *ν*(C–H), of disubstituted *cis* double bonds, as those found in the unsaturated chains of linseed oil.^35^ Antisymmetric and symmetric C–H stretching modes of the methylene groups of the fatty acid chains can be observed at 2922 and 2853 cm^−1^, respectively. Bands characteristic of C–H vibrational modes of terminal methyl (CH_3_) groups were not observed between 3000 and 2800 cm^−1^, which indicates a high proportion of CH_2_/CH_3_. This information is according to the presence of the long chain fatty acids of linseed oil. The strong band at 1739 cm^−1^ is assigned to C=O stretching modes, *ν*(C=O), of carbonyl groups characteristic of esters. Esters present also C–O stretching modes, *ν*(C–O), between 1300 and 1100 cm^−1^. The spectrum in the Fig. 8 shows a broad unsolved envelope of bands, which can include C–O ester vibrations around 1165 cm^−1^.^35^

The bands characteristic of LC-PUFAs shown in the spectrum of the Fig. 7 indicates the functional groups of linseed oil were preserved during the glycerolysis reaction. Besides, the high proportion of hydroxyl and methylene groups indicate MAG was successful synthesized from linseed oil.

Fig. 8 shows the TGA curves, of the biodiesel obtained with 97% yield, and monoacylglycerol, obtained with 76% yield. The biodiesel thermogram shows a weight loss of approximately 96% in only one-step. This thermal event occurred between 160 and 260 °C with maximum weight loss at 244 °C, which can be attributed to the degradation of organic matter.

On the other hand, the MAG presents a different thermal decomposition profile. The thermogram in the Fig. 8 shows a weight loss of approximately 78% and, at least, three thermal events between 160 and 510 °C. These thermal events can be observed by the different slopes in the curve. Besides, thermal decomposition of MAG seems to be slower than shown by biodiesel. It should be considered that MAG structure has hydroxyl groups, which can significantly interfere in its thermal decomposition mechanism.

## 4. Conclusion

The results obtained in our experiments indicate the production feasibility of monoacylglycerol through the glycerolysis of ethyl ester mixture (biodiesel). Also, we can obtain high-purity ethylic esters by the TDSP method, with conversion of 97%. With respect to the system, it is demonstrated that the most efficient one was the open reactor than the reflux reactor. Monoglycerides were produced at high yields and selectivities. Conversions (defined as the percentage of consumed fatty acid) reached 98%, with monoglyceride being the major product, with yield of 76%. Thus, we can achieve expressive results for green monoacylglycerol production through a cheaper chemical route possible for several industrial sectors like the food and pharmaceutical. Finally, it should be stressed that the production of monoglycerides was successfully carried out in a very simple reaction system.

## List of abbreviations

A (CAL-A): *Candida antarctica* lipase
ASTM: American society for testing and materials
DAG: Diacylglycerol
FAEE: Fatty acid ethyl ester
FAME: Fatty acid methyl esters
IL: Ionic liquid
LC-PUFA: Long-chain polyunsaturated fatty acid
MAG: Monoacylglycerol
MSTFA: *N*-Methyl-*N*-(trimethylsilyl)trifluoroacetamide
MUFA: Monounsaturated fatty acid
*n* − 3PUFA: *n* − 3 Polyunsaturated fatty acid
PUFA: Polyunsaturated fatty acid
SFA: Saturated fatty acid
TAG: Triacylglycerol
TDSP: Transesterification double step process

## Conflicts of interest

There are no conflicts to declare.

## Supplementary Material

## References

[cit1] Slobodian P., Vícha R., Doležálková I. V. A., Janiš R., Bun L. (2013). J. Food Biochem..

[cit2] Isaacs C. E., Litov R. E., Thormars H. (1995). J. Nutr. Biochem..

[cit3] Bunka F., Pavlínek V., Hrabě J., Rop O., Janis R., Krejci J. (2007). Int. J. Food Prop..

[cit4] Christie W. W. and Han X., Lipid analysis: isolation, separation, identification and lipidomic analysis, Oily Press, Washington, 2010

[cit5] Noureddini H., Medikonduru V. (1997). J. Am. Oil Chem. Soc..

[cit6] Luo H., Zhai Z., Fan W., Cui W., Nan G., Li Z. (2015). Ind. Eng. Chem. Res..

[cit7] Sonntag N. O. V. (1982). J. Am. Oil Chem. Soc..

[cit8] Schulz A. S., Silveira K. C., Libardi D. B., Peralba C. R. (2011). Eur. J. Lipid Sci. Technol..

[cit9] He Y., Li J., Kodali S., Chen B., Guo Z. (2016). Bioresour. Technol..

[cit10] Lozano P., Gomez C., Nieto S., Sanchez-Gomez G., Garcia-Verdugo E., Luis S. V. (2017). Green Chem..

[cit11] Kulkarni M. G., Gopinath R., Meher L. C., Dalai A. K. (2006). Green Chem..

[cit12] Drapcho C. M. , Nghim N. P. and Walker T., Biodiesel, in Biofuels Engineering Process Technology, McGraw-Hill, 2008

[cit13] Pinzi S., Garcia I. L., L. De Castro M. D., Dorado G., Dorado M. P. (2009). Energy Fuels.

[cit14] Lotero E., Liu Y., Lopez D. E., Suwannakarn K., Bruce D. A., Goodwin J. G. (2005). Ind. Eng. Chem. Res..

[cit15] Černoch M., Hájek M., Skopal F. (2010). Bioresour. Technol..

[cit16] Stamenkovic O. S., Velickovic A. V., Veljkovic V. B. (2011). Fuel.

[cit17] Bancquart S., Vanhove C., Pouilloux Y., Barrault J. (2001). Appl. Catal. Gen..

[cit18] Ho D. P., Hao H., Guo W. (2014). Bioresour. Technol..

[cit19] Karmakar A., Karmakar S., Mukherjee S. (2010). Bioresour. Technol..

[cit20] Zambiazi R. U. I. C., Przybylski R., Zambiazi M. W., Mendonça C. B. (2007). Bol. Cent. Pesqui. Process. Aliment..

[cit21] Huang J., Wang Q., Li T., Xia Q. (2018). J. Sci. Food Agric..

[cit22] Czlonka S., Bertino M. F., Kosny J., Strakowska A., Maslowski M., Strzelec K. (2020). Ind. Crops Prod..

[cit23] Jebrane M., Cai S., Sandström C., Terziev N. (2017). eXPRESS Polym. Lett..

[cit24] Freedman B., Pryde E. H., Mounts T. L., Regional N. (1984). J. Am. Oil Chem. Soc..

[cit25] Corma A., Bee S., Hamid A., Iborra S., Velty A. (2005). J. Catal..

[cit26] Nelson L. A., Foglia T. A., Marmer W. N. (1996). J. Am. Oil Chem. Soc..

[cit27] Shimada Y., Watanabe Y., Sugihara A., Tominaga Y. (2002). J. Mol. Catal. B: Enzym..

[cit28] Kumar R., Tiwari P., Garg S. (2013). Fuel.

[cit29] Samios D., Pedrotti F., Nicolau A., Reiznautt Q. B., Martini D. D., Dalcin F. M. (2009). Fuel Process. Technol..

[cit30] Demirbas A. (2003). Energy Convers. Manage..

[cit31] Fukuda H., Kond A., Noda H. J. (2001). J. Biosci. Bioeng..

[cit32] Basso R. C., Meirelles A. J. A., Batista E. A. C. (2017). Braz. J. Chem. Eng..

[cit33] Neto O. Z. S., Batista E. A. C., Meirelles A. J. de A. (2018). J. Cleaner Prod..

[cit34] Kaur N., Ali A. (2015). RSC Adv..

[cit35] Guzatto R., Defferrari D., Reiznautt Q. B., Cadore Í. R., Samios D. (2012). Fuel.

[cit36] Dias T. P. V. B., Neto P. M., Ansolin M., Follegatti-Romero L. A., Batista E. A. C., Meirelles A. J. A. (2015). Braz. J. Chem. Eng..

[cit37] Tu Q., Lu M., Knothe G. (2017). J. Cleaner Prod..

[cit38] Díaz-álvarez A. E., Francos J., Crochet P., Cadierno V. (2014). Green Chem..

[cit39] Gelbard G., Bres O., Vargas R. M., Vielfaure F., Schuchardt U. F. (1995). J. Am. Oil Chem. Soc..

[cit40] Knothe G., Kenar J. A. (2004). Eur. J. Lipid Sci. Technol..

[cit41] Morgenstern M., Cline J., Meyer S., Cataldo S. (2006). Energy Fuels.

[cit42] Tariq M., Ali S., Ahmad M., Zafar M. (2011). Fuel Process. Technol..

[cit43] Lenardão E. J., Dabdoub M. J., Batista C. F. (2003). Quim. Nova.

[cit44] Bakare I. O., Pavithran C., Okieimen F. E., Pillai C. K. S. (2006). J. Appl. Polym. Sci..

